# Clinical Features and Prognostic Factors of Acute Ischemic Stroke Related to Malignant Gastrointestinal Tumor

**DOI:** 10.3389/fneur.2021.777483

**Published:** 2021-11-25

**Authors:** Yating Liu, Xin Li, Feixue Song, Xin Yan, Zhijian Han, Futian Tang, Yumin Li

**Affiliations:** ^1^Department of Oncology, Lanzhou University Second Hospital, Lanzhou, China; ^2^Key Laboratory of the Digestive System Tumors of Gansu Province, Lanzhou University Second Hospital, Lanzhou, China; ^3^Department of Neurology, Lanzhou University Second Hospital, Lanzhou, China

**Keywords:** gastrointestinal malignant tumor, acute ischemic stroke, D-dimer (DD), TAT, prognosis

## Abstract

**Objectives:** To analyze the clinical and imaging features of acute ischemic stroke (AIS) related to gastrointestinal malignant tumor, and to explore the prognostic factors.

**Methods:** Clinical data of consecutive patients with gastrointestinal malignant tumor complicated with AIS admitted to the Department of Neurology and Oncology in Lanzhou University Second Hospital from April 2015 to April 2019 were retrospectively analyzed. Patients were divided into good prognosis (mRS 0–2) and poor prognosis (mRS > 2) based on a 90-day mRS score after discharge. The multivariate logistic regression model was used to analyze the prognostic factors.

**Results:** A total of 68 patients were enrolled with an average age of 61.78 ± 6.65 years, including 49 men (72.06%). There were 18 patients in the good prognosis group and 50 patients in the poor prognosis group. The univariate analysis showed that Hcy, D-dimer, thrombin–antithrombin complex (TAT), and three territory sign in magnetic resonance imaging (MRI) were the risk factors for poor prognosis. Multivariate analysis showed that increased D-dimer (OR 4.497, 95% CI 1.014–19.938) and TAT levels (OR 4.294, 95% CI 1.654–11.149) were independent risk factors for the prognosis in such patients.

**Conclusion:** Image of patients with gastrointestinal malignant tumor-related AIS is characterized by three territory sign (multiple lesions in different vascular supply areas). Increased TAT and D-dimer levels are independent prognostic risk factors. TAT is more sensitive to predict prognosis than D-dimer.

## Introduction

Armand Trousseau first reported the relation between thrombosis and malignant tumors in 1865 (1). Subsequent studies have confirmed that thrombosis is a common complication of malignant tumors, which is the second leading cause of death in patients with cancer (2). Thrombotic complications of cancer include arterial or venous thromboembolism and disseminated intravascular coagulation (DIC) (3, 4). Up to 15% of patients with malignant tumors have a history of acute ischemic stroke (AIS) (5), and about 20% of patients with AIS due to an unknown cause (cryptogenic stroke) may have latent malignant tumors (6). Previous studies on gastrointestinal malignant tumor-related thromboembolism mainly focused on venous thromboembolism (7), and clinical studies on the associated AIS focused on a single type of gastrointestinal tumor (8), leading to a paucity of systematic analysis of different gastrointestinal tumor types. The present work retrospectively analyzes the 5-year clinical data of consecutive patients with gastrointestinal malignant tumor-related AIS in a single center. Prognostic factors are analyzed to improve clinical understanding of the gastrointestinal malignant tumor-related AIS.

## The Mechanisms of Malignant Tumor-Related AIS

Abnormal coagulation function is the main cause of AIS in patients with malignant tumor. Abnormal cerebrovascular coagulation is seen in patients with malignant tumors including breast cancer, leukemia, and lymphoma, after performing autopsy, which indicated the association between malignant tumors and coagulation and thrombosis (9). Kim et al. (10) conducted a prospective study and found that D-dimer levels in tumor-related cerebral infarction patients were significantly higher with increased incidence of multifocal cerebral infarction compared with conventional stroke risk factors. Tumor onset and progression are often accompanied by hypercoagulability, resulting in systemic and cerebral–arterial or venous thrombosis (11).

**Figure 1:**
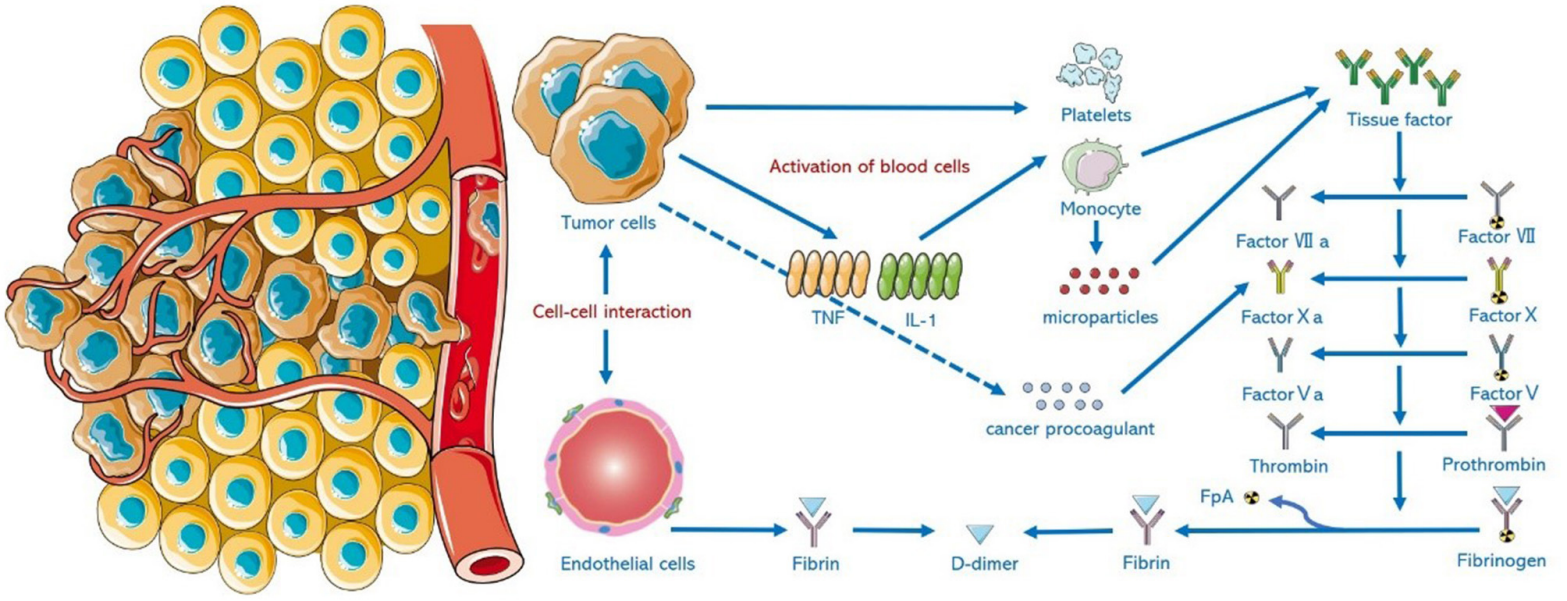
Mechanism of hypercoagulable state in malignant tumors. Tumor cells activate cellular systems *in vivo* through intercellular interactions and injured endothelial cells. Tumor cells can directly release tissue factor (TF) and cancer procoaparticles (CP). Tumor cells produce cytokines including interleukin-1 (IL-1) and tumor necrosis factor (TNF). Activated blood cells (such as monocytes and platelets) and the microparticles (MP) produced by these cells work synergistically to increase TF expression and activate the coagulation system *in vivo*.

Adenocarcinomas, especially those of the pancreas, colon, breast, lung, prostate, and ovary induce thrombosis by producing and releasing mucin (a high-molecular weight molecule that is glycosylated and secreted by the endothelial cells) directly into the blood, which promotes a hypercoagulation state. Mucin can also interact with certain cellular adhesion molecules on endothelial cells, platelets, and lymphocytes, triggering the formation of microthrombi, which is rich in platelets (12). Meanwhile, tumor cells can produce cancer coagulants (cysteine protease that can independently activate coagulation X factor), tissue factors (binding with coagulation factor VII), and release inflammatory and vascular endothelial growth factors that can mediate coagulation and thrombosis (13–15). For example, tumor necrosis factor-α (TNF-α) affects the anticoagulant properties of the vascular endothelial cells through tissue factors, which promotes thrombin production, fibrin clot formation, and fibrin deposition in blood vessels, and can reduce fibrinolysis by inhibiting tPA activity (16). Tumors may also be associated with acute or chronic DIC. Due to excessive activation of the coagulation process, the imbalance between coagulation and fibrinolysis will eventually lead to thrombosis and arterial occlusion, resulting in AIS (17). The binding of malignant tumor cells with certain nonspecific immune signaling molecules, such as selectin, chemokines, and the corresponding receptors, is conducive to tumor invasion, migration, and adhesion. This will damage cellular connection and cause endothelial injury (18). The activation of the host immune system and the release of inflammatory factors will damage vascular endothelium and thus promote thrombosis (19). Figure 1 shows the formation mechanism of hypercoagulable state in malignant tumors.

Nonbacterial thrombotic endocarditis (NBTE) caused by platelet thrombotic inflammatory complex is another important cause of malignant tumor-related AIS. In a work which consisted of 2,627 cases of postmortem examination, found that in 16 patients with malignant tumor complicated with NBTE, seven were complicated with cerebral infarction (20). This indicated that detaching of cardiac embolus in patients with malignant tumor complicated with NBTE can directly lead to AIS. In addition, infection (21, 22), tumor-related chemotherapy (23), endocrine therapy (24, 25) and, radiotherapy (26) can also lead to AIS in patients with a malignant tumor.

## Materials and Methods

### Study Population

The clinical baseline data of patients with gastrointestinal malignant tumor complicated with AIS admitted to the Department of Neurology and Oncology in Lanzhou University Second Hospital from April 2015 to April 2019, were retrospectively analyzed. Eligible patients were required to meet the following criteria: (1) patients aged ≥18 years; (2) diagnosed with acute cerebral infarction conforming to Chinese Guidelines for the Diagnosis and Treatment of Acute Ischemic Stroke (2014) (5); (3) having clear imaging evidence in magnetic resonance imaging (MRI); (4) within 7 days of onset; (5) with a history of active malignant gastrointestinal tumor in the past or during follow-up. Patients were excluded if they had the following characteristics: (1) a history of TIA or cerebral hemorrhage; (2) primary tumors of the central nervous system, hematological, and other systems; (3) incomplete medical records; (4) poor prognosis due to tumor progression or chemotherapy.

### Study Measures

#### Baseline and MRI Data

General information of the patients was retrieved, including gender, age, medical history and complications, laboratory tests on the second day of admission, tumor related data, and the results of echocardiography. The MRI data of the patients were collected. The location and the number of lesions on DWI were recorded. Lesions in both hemispheres were defined as bilateral infarction, and multiple infarctions referred to more than two lesions. Distribution patterns of the lesions were categorized into the following: (1) one territory sign: single or multiple lesions in unilateral anterior or posterior circulation; (2) two territory sign: single or multiple lesions in unilateral anterior and posterior circulations, or in bilateral anterior circulations; (3) three territory sign: bilateral single or multiple lesions in bilateral anterior and posterior circulations. Prognosis of the patients was evaluated by mRS, 90 days after discharge. An mRS score of 0–2 was defined as good prognosis, and score >2 was regarded as poor prognosis.

#### Statistical Analysis

Statistical analysis was performed by the SPSS 25.0. Enumeration data were recorded in ratio or proportion using χ2 or Fisher tests for comparisons among groups. Normally distributed measurement data was expressed as X ± S, using *t*-test for comparisons among groups. Median and range (M, P25–P75) were used if the measurement data were not normally distributed, and the Mann–Whitney U test was used for comparisons among groups. Variables with *P* < 0.05 in univariate analysis were taken as independent variables with a 90-day prognosis as dependent variable. Multivariate logistic regression analysis was used to screen out independent risk factors for prognosis. *P* < 0.05 was considered statistically significant.

## Results

**Figure 2:**
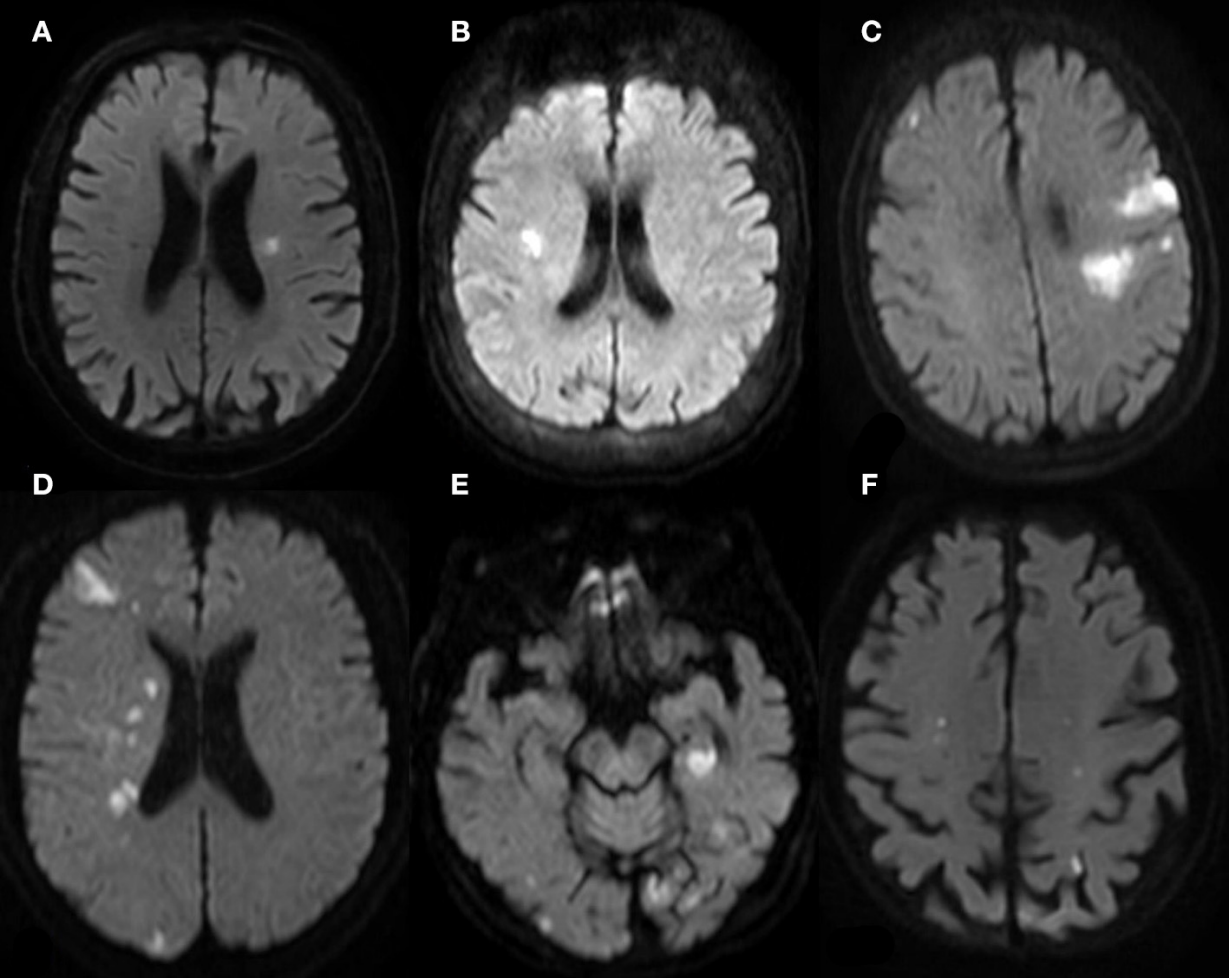
MR (DWI) of patients with gastrointestinal malignant tumor-related AIS. **(A,B)** One Territory Sign (different patients): lesions with hyperintense signal in the supply area of LMCA in **(A)**; lesions with hyperintense signal in the supply area of RMCA. **(C,D)** Two Territory Sign (different patients): multiple lesions in the supply area of LMCA and RACA in **(C)**, involving cortex and deep white matter region; multiple lesions in the supply area of RMCA and RPCA in **(D)**. **(E,F)** Three Territory Sign (the same patient): massive lesions in the left posterior hippocampus region supplied by the posterior choroid artery of LPCA; scattered lesions in bilateral occipital cortex and subcortex, more prominent on the left side. **(E)** shows two dotted lesions in the supply area of bilateral internal carotid arteries.

A total of 68 patients with gastrointestinal cancer complicated with AIS were enrolled in this study, including 49 (72.09%) men and 19 (27.91%) women, with a mean age of 61.78 ± 6.65 years. Cases of complications included hypertension, diabetes, hyperlipidemia, and coronary heart disease, and were 45 (66.18%), 38 (55.88%), 43 (63.24%), and 45 (66.18%), respectively. Patients with a history of drinking and smoking accounted for 50.00 and 35.29%, respectively. Fifteen (22.06%) cases and 14 (20.59%) cases had a history of atrial fibrillation and stroke, respectively. The types of gastrointestinal malignant tumors included gastric cancer (42/68, 61.76%), colorectal cancer (17/68, 25.00%), gallbladder cancer (4/68, 5.88%), pancreatic cancer (2.68, 2.94%), liver cancer (2/68, 2.94%), and esophageal cancer (1/68, 1.47%). Adenocarcinoma was the main pathological tumor type, accounting for 63/68 cases (92.65%). Fifty-two (76.47%) cases had a history of malignant tumor, 16 (23.53%) cases without a previous history of tumor. Malignant tumors were diagnosed during hospitalization or follow-up due to AIS. MRI showed multiple scattered lesions. Patients with two and three territory signs accounted for 25 (36.76%) and 30 (44.12%) cases, respectively; one territory sign was only observed in seven (10.29%) cases. Figure 2 shows the characteristics of gastrointestinal malignant tumor-related AIS in MRI.

The baseline characteristics of all study population and univariate regression analysis of prognosis are summarized in Table 1, with 18 patients in the good prognosis group and 50 patients in the poor prognosis group. Compared with the good prognosis group, patients in the poor prognosis group of three territory sign in MRI (52.00 vs. 22.22%, *P* = 0.029) showed higher levels of Hcy (8.84 ± 3.03 vs. 17.00 ± 3.40 μmol/L, *P* = 0.047) and D-dimer (2.12 ± 0.78 vs. 1.24 ± 0.70 mg/L, *P* < 0.001), and also higher levels of TAT (8.07 ± 3.99 vs. 3.75 ± 1.40 ng/ml, *P* < 0.001), all with significant differences (*P* < 0.05).

**Table 1:** Univariate regression analysis of poor prognosis in patients with gastrointestinal malignant tumor-related AIS.

**Item**	**Total (68)**	**Good prognosis (18)**	**Poor prognosis (50)**	***P*-value**
Male, *n* (%)	49 (72.06)	14 (77.78)	35 (70.00)	0.528
Age, years	61.78 ± 6.65	61.78 ± 6.11	61.78 ± 6.90	0.741
Hypertension, *n* (%)	45 (66.18)	9 (50.00)	36 (72.00)	0.091
Diabetes, *n* (%)	38 (55.88)	7 (38.89)	31 (62.00)	0.090
Hyperlipidemia, *n* (%)	43 (63.24)	9 (50.00)	34 (68.00)	0.174
AF, *n* (%)	15 (22.06)	2 (11.11)	13 (26.00)	0.330
CHD, *n* (%)	37 (54.41)	8 (44.44)	29 (58.00)	0.322
Stroke, *n* (%)	14 (20.59)	4 (22.22)	10 (20.00)	1.000
Smoking, *n* (%)	24 (35.29)	6 (33.33)	18 (36.00)	0.839
Drinking, *n* (%)	34 (50.00)	10 (55.56)	24 (48.00)	0.582
Previous tumor history, *n* (%)	52 (76.47)	11 (61.11)	41 (82.00)	0.574
MT to stroke after tumor, month	6.15 ± 3.21	6.86 ± 2.26	5.64 ± 3.02	0.812
Diagnosis after stroke, *n* (%)	16 (23.53)	7 (38.89)	9 (18.00)	0.871
MT to tumor after stroke, month	12.39 ± 4.12	13.21 ± 3.22	10.18 ± 2.41	0.072
RBC (×1012/L)	4.51 ± 0.75	4.62 ± 0.90	4.47 ± 0.69	0.833
WBC (×109/L)	7.01 ± 2.10	7.76 ± 2.02	6.74 ± 2.08	0.810
PLT (×109/L)	314.22 ± 20.85	306.33 ± 29.53	317.06 ± 16.14	0.326
Hb, g/L	119.09 ± 10.34	122.56 ± 15.30	117.84 ± 7.69	0.231
LDL-C, mmol/L	4.11 ± 0.72	3.89 ± 0.70	4.19 ± 0.72	0.130
Hcy, μmol/L	18.35 ± 3.21	17.00 ± 3.40	18.84 ± 3.03^a^	0.047
Fib, g/L	4.30 ± 0.60	4.18 ± 0.57	4.34 ± 0.61	0.981
D-dimer, mg/L	1.89 ± 0.85	1.24 ± 0.70	2.12 ± 0.78^*a*^	<0.001
TAT, ng/ml	6.93 ± 3.98	3.75 ± 1.40	8.07 ± 3.99^*a*^	<0.001
One territory sign	7 (10.29)	4 (22.22)	3 (6.00)	0.136
Two territory sign	25 (36.76)	4 (22.22)	21 (42.00)	0.136
Three territory sign	30 (44.12)	4 (22.22)	26 (52.00)^*a*^	0.029
Cardiac valvular vegetations	13 (19.12)	2 (11.11)	11 (22.00)	0.511

*CHD, coronary heart disease; AF, atrial fibrillation; MT, mean time; RBC, Red blood cell; WBC, White blood cell; PLT, Platelet; Hb, Hemoglobin; LDL-C, low-density lipoprotein cholesterol; Hcy, homocysteine; Fib, fibrinogen; TAT, thrombin-antithrombin complex*.

a *P < 0.05, as compared to poor prognosis*.

Table 2 shows the multivariate analysis of prognosis. The results showed higher levels of D-dimer (OR 4.497, 95% CI 1.014–19.938) and TAT (OR 4.294, 95% CI 1.654–11.149) which are independent risk factors for the prognosis in patients with gastrointestinal malignant tumor-related AIS (*P* < 0.05).

**Table 2:** Multivariate analysis of poor prognosis in patients with gastrointestinal malignant tumor-related AIS.

**Variables**	**β**	**SE**	**Wals**	**Df**	***P*-value**	**OR**	**95% CI**
Hcy	0.259	0.164	2.493	1	0.114	1.296	0.939–1.788
D-dimer	1.503	0.76	3.914	1	0.048	4.497	1.014–19.938
TAT	1.457	0.487	8.957	1	0.003	4.294	1.654–11.149
Three territory sign	2.2	1.216	3.271	1	0.071	9.021	0.832–97.827

**Figure 3:**
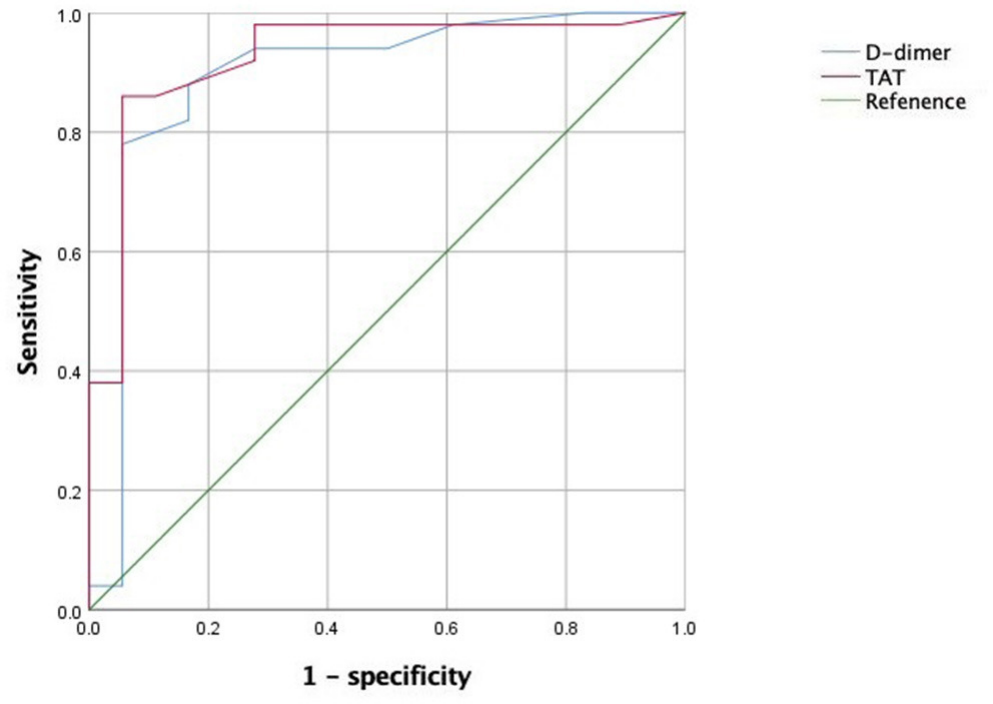
ROC curve of TAT and D-dimer levels for prognosis in patients with gastrointestinal malignant tumor-related AIS.

The baseline levels of D-dimer and thrombin–antithrombin (TAT) were 1.89 ± 0.85 and 6.93 ± 3.98 ng/ml, respectively. The levels of D-dimer at 3, 6, and 9-month follow-up were 1.65 ± 0.80, 1.52 ± 0.97, and 1.39 ± 1.02 mg/L, respectively; the levels of TAT were 8.46 ± 6.17, 9.88 ± 8.61, and 9.33 ± 8.82 ng/ml, respectively. An ROC curve analysis showed that when the AUC of D-dimer was 0.894 with a cutoff value of 1.60 mg/L FEU, the diagnostic sensitivity and specificity were 78.0 and 94.4%, respectively; when the AUC of TAT was 0.926 with a cutoff value of 5.05 ng/mL, the diagnostic sensitivity and specificity of TAT were 86.0 and 94.4%, respectively. Figure 3 show ROC curve of TAT and D-dimer levels for prognosis in patients with gastrointestinal malignant tumor-related AIS.

## Discussion

Acute ischemic stroke may be identified prior to malignant tumor, which may be an early evidence of potential malignant tumor. Previous studies have found that patients with malignant tumor and with AIS as an initial symptom accounted for 0.4–3.0% of hospitalized stroke patients, and about 5.3–20% of latent stroke patients were diagnosed with malignant tumor during hospitalization, commonly including lung, breast, liver, stomach, and prostate cancers (27, 28). Malignant tumor-related AIS is prone to early neurological deterioration, with high recurrence and poor prognosis, and the prognosis varies in patients with different types of such cerebral infarctions (29). Based on the 2020 data from the International Agency for Research on Cancer (IARC), colorectal, liver, and gastric cancers were the third to fifth common malignant tumors in men, and colorectal cancer is the second common cancer in women (30). Analyzing the characteristics of gastrointestinal malignant tumor-related AIS is the basis for the treatment of such kinds of disease.

### Prognostic Factors in Patients With Gastrointestinal Malignant Tumor-Related AIS

The results showed no significant difference between good and poor prognosis groups in common risk factors of AIS including hypertension, coronary heart disease, diabetes, and hyperlipidemia. Higher levels of fibrinogen, TAT, and D-dimer, and greater proportion of patients with multiple scattered infarctions were observed in the poor prognosis group, which indicated a different mechanism of tumor-related AIS from the common atherosclerotic infarction. It has been proposed that tumor necrosis factors or cytokines produced by tumor cells can promote coagulation through DIC and form microthrombi (31). The mechanisms of malignant tumor-related AIS include hypercoagulability, NBTE, infection, tumor-related chemotherapy, endocrine therapy, and radiotherapy, among which, hypercoagulability plays the most important role. This study found that increased levels of D-dimer and TAT are independent prognostic factors of such patients, which is consistent with the results of previous studies (32). Results of this study also confirmed that hypercoagulation may be the major mechanism of cerebral infarction in patients with a malignant tumor.

### TAT as a Better Prognostic Factor Than D-Dimer in Tumor-Related AIS

D-dimer has been proposed as a predictor of AIS in patients with a malignant tumor (33, 34). However, the limitations of the testing methods, interference by rheumatoid factor, immune complex, and intake of antitumor drugs or immunoenhancing drugs may lead to false positive results (35). With the updated testing methods, TAT and other indicators become more suitable for clinical application. These indicators are molecular markers of thrombosis and fibrinolysis that can reflect the state of coagulation and fibrinolysis in patients with a malignant tumor (36, 37). TAT is a complex formed by thrombin and antithrombin III. As a molecular marker of thrombin formation, TAT directly reflects the activation of the coagulation system. Increased TAT is a hint of procoagulant activation and inhibitor depletion and is one of the earliest indicators of coagulation dysfunction (38). Fidan et al. found that the level of TAT in gastric cancer patients was significantly higher than that in the control group, with stable expression at different tumor stages, which was a reliable marker for the hypercoagulation state of malignant tumors (39). The present study also confirmed that TAT expression was significantly correlated with the clinical prognosis in patients with gastrointestinal malignancy, and was more sensitive and specific than D-dimer. Therefore, we believe that TAT is better than D-dimer in predicting the prognosis of patients with malignant tumor-related thrombotic events, which is consistent with the results of Cui et al. (40).

### MRI Features of Gastrointestinal Malignant Tumor-Related AIS

Previous research found that in AIS patients complicated with malignant tumor the incidence of infarction simultaneously involving three main intracranial arterial supply areas was six times that of patients with atrial fibrillation (41). A significant increase of D-dimer is a characteristic of malignant tumor-related AIS (42–44). In this study, most patients showed infarction in three territories on DWI (44.12%), which was an independent risk factor for prognosis.

Echocardiogram was performed in all the patients to identify potential cardiogenic embolism, and 19.12% (13/68) of the patients were found to have NBTE which is basically consistent with the proportion of NBTE in about 19% of patients with malignant tumors found by Edoute et al. (45). NBTE is most common in lung or gastrointestinal adenocarcinoma-related AIS associated with lung cancer or gastrointestinal adenocarcinoma, which is also reported in pancreatic and biliary cancer (46). However, NBTE was not found in patients with pancreatic and biliary cancer in this study.

### Prognosis, the Temporal Relation Between Gastrointestinal Malignant Tumor, and AIS

It has been reported that the mortality of AIS was higher in patients complicated with malignant tumor than those without the tumor (47). The former research showed that a 3-month mortality rate of 46.9% in patients with malignant tumor complicated with AIS after receiving relevant treatment and 50% of the survivors had neurological sequela (48). Another study showed that an average follow-up of 29 months for 24 patients with cerebral infarction complicated with latent tumor, with mortality reaching 79%. At a 1-year follow-up, 73.53% (50/68) of the patients showed poor outcomes (mRS > 2) including deaths (27). The poor therapeutic effects may be attributed to the complex mechanisms of cancer, or the onset of AIS leading to aggravation of the general condition of the patients, even affecting, or interrupting antitumor therapy.

Selvik et al. (49) found an average interval of 14 months from the onset of AIS, to the diagnosis of the tumor. In the present study, 16 patients (23.53%) were diagnosed with malignant gastrointestinal tumor 12.39 ± 4.12 months after AIS; 52 patients (76.47%) developed AIS after the diagnosis of tumor, with an average interval of 6.15 ± 3.21 months, which is basically consistent with previous results showing an interval of 1.5–9 months (50).

## Conclusions

Patients with AIS related to gastrointestinal malignant tumor have a relatively poor prognosis. Increased levels of TAT and D-dimer are independent risk factors for poor prognosis. TAT has a better performance than D-dimer in predicting prognosis in patients with gastrointestinal malignant tumor-related AIS.

## Limitations

Some limitations have to be admitted for we report a retrospective study with a relatively small number of patients. Due to the limited number of the included cases, tumor stage and treatment were not analyzed, which could limit the statistical significance of the results. Multicenter studies and larger sample are required for analysis to better summarize the clinical characteristics of malignant tumor patients complicated with AIS in future.

## Data Availability Statement

The raw data supporting the conclusions of this article will be made available by the authors, without undue reservation.

## Ethics Statement

The studies involving human participants were reviewed and approved by Lanzhou University Second Hospital Ethics Committee. The patients/participants provided their written informed consent to participate in this study. Written informed consent was obtained from the individual(s) for the publication of any potentially identifiable images or data included in this article.

## Author Contributions

YLiu and XL contributed to the study design and drafted the manuscript. FS and XY analyzed the data in the literature and edited the manuscript. YLi contributed to the study design, supervised writing of the manuscript, critically reviewed the paper, and supervised the research project. All authors contributed to the article and approved the submitted version.

## Funding

This work was supported by grants from the Science and Technology Foundation for Young Scientist of Gansu Province, China (Grant no. 21JR1RA163), The Natural Science Foundation of Gansu Province, China (Grant no. 21JR1RA136), The Science and Technology Project of CuiYing, Lanzhou University Second Hospital, China (Grant no. CY2018-MS06), and Major Science and Technology Projects of Gansu Province, China (20ZD7FA003).

## Conflict of Interest

The authors declare that the research was conducted in the absence of any commercial or financial relationships that could be construed as a potential conflict of interest.

## Publisher's Note

All claims expressed in this article are solely those of the authors and do not necessarily represent those of their affiliated organizations, or those of the publisher, the editors and the reviewers. Any product that may be evaluated in this article, or claim that may be made by its manufacturer, is not guaranteed or endorsed by the publisher.
